# Notes and News

**Published:** 1906-04-28

**Authors:** 


### Notes and News.

Lady Northcote, wife of the Governor-General of the Australian Commonwealth, has presented half a dozen children's cots to the Sutherland Home at Melbourne.

The United States Ambassador, Mr. Whitelaw Reid, will preside at the festival dinner in aid of the Charing Cross Hospital Emergency, to be held at the Hotel Cecil on Wednesday, May 23.

The first dental surgeons' exhibition ever held in London is now open at the Cavendish Rooms, Mortimer Street, and a number of the latest dental materials, instruments, and inventions are on view.

On Friday, at Wimborne, the Duke of Hamilton applied for and obtained a vaccination exemption certificate on the ground that he conscientiously believes vaccination would be prejudicial to the health of his child.

Under the will of Mrs. Elizabeth Storer, widow of Dr. Charles Storer, of Lowdham Grange, Notts, the sum of £2,000 is bequeathed to the Nottingham General Hospital to endow beds in memory of Dr. Storer and his wife.

An influential committee of American ladies residing in England is being organised with a view of showing their great sympathy with the sufferers in the San Francisco disaster by raising subscriptions towards rebuilding one of the great charitable institutions which have been destroyed. Among those who have already joined are Mrs. Chamberlain, Lady Curzon, Mrs. Adair, the Duchess of Marlborough, Mrs. Ronalds, and Mrs. Lewis Harcourt.

At the Elizabethan Fair and Fête in aid of the King's College Hospital Removal Fund, to be held on May 23 and two following days at Lincoln's Inn, the stalls in the grounds will represent Old Cheapside, the Old Rye House, and the Market Cross at Enfield.

The success of the work carried on at the Nordrach-on-Dee Sanatorium for the Open-air Treatment of Consumption was announced at the recently held annual meeting. This sanatorium is situated in the pine district of Middleside, near Balmoral, and is regarded as one of the most completely equipped private institutions in existence, while its fame has drawn to it patients from all parts of the United Kingdom, the Colonies, and other distant lands. The institution is fortunate in having secured the services of Dr. Lawson, the medical superintendent, for a further five years. His system of treatment has been approved and adopted by the Aberdeen Medico-Chirurgical Society.

The idea of utilising old horse tramway-cars for consumptive patients has been carried into practical operation in Leith. In a field with a southern exposure near the Pilton Hospital for Infectious Diseases four old cars have been stationed. Very little has been done to them. Merely the window glass has been knocked out on the south side, and one of the seats fitted up for two bunks. Not a penny has been spent in painting or in any unnecessary work. On the top of the cars the fixed seats are cleared off, and garden chairs placed ready for the patients when the weather is sufficiently favourable to allow of them sitting without shelter. As the kitchens of the Pilton Hospital are at no distance away there is no trouble in regard to cooking, etc.

On Monday a new ophthalmic department, consisting of two wards and a theatre, was opened by Lady Belper at the Derbyshire Royal Infirmary. All the thirty beds, furniture, etc., are new, the building and general arrangements have been carried out on the most modern principles, and the wing is lighted throughout by electricity. The honorary ophthalmic surgeon to the infirmary having briefly described the necessity for the new wards, and Lady Belper having declared them open, Lord Belper, acknowledging on behalf of his wife a vote of thanks, referred to the generosity of the late Mr. Henry Evans, whose bequest had provided for the building and equipment of the new block.

